# Enhanced Sub-ppm NH_3_ Gas Sensing Performance of PANI/TiO_2_ Nanocomposites at Room Temperature

**DOI:** 10.3389/fchem.2018.00493

**Published:** 2018-10-18

**Authors:** Chonghui Zhu, Xiaoli Cheng, Xin Dong, Ying ming Xu

**Affiliations:** Key Laboratory of Functional Inorganic Material Chemistry, Ministry of Education, School of Chemistry and Materials Science, Heilongjiang University, Harbin, China

**Keywords:** gas sensing, polyaniline, TiO_2_, NH_3_, nanocomposites

## Abstract

PANI/TiO_2_ nanocomposites spheres were synthesized using a simple and efficient one-step hydrothermal process. The morphology and structure of PANI/TiO_2_ nanocomposites spheres were investigated by X-ray diffraction (XRD), scanning electron microscopy (SEM), and transmission electron microscopy (TEM) techniques. The PANI/TiO_2_ nanocomposite sphere-based sensor exhibits good selectivity, sensitivity (5.4 to 100 ppm), repeatability, long-term stability and low detection limit (0.5 ppm) to ammonia at room temperature (20 ± 5°C). It also shows a good linearity relationship in the range of 0.5–5 and 5–100 ppm. The excellent NH_3_ sensing performance is mainly due to the formation of the p-n heterostructure in the nanocomposites.

## Introduction

Polyaniline (PANI), as an intrinsically conductive polymer, is the widely used material in anti-corrosion, anti-static electricity, stealth and so on. Since its low density, excellent processability, excellent flexibility, and good electrical conductivity, (Bhadra et al., 2009; Baker et al., 2017) polyaniline is also attracting more attention in the field of gas sensing. However, pure PANI sensors often show lower sensitivity or higher detection limit than semiconductor metal oxide sensors (Sutar et al., 2007; Kebiche et al., 2012). The addition of semiconducting metal oxides not only improves gas-sensing properties, but also avoids high operating temperatures. In recent years, SnO_2_, ZnO, TiO_2_, and In_2_O_3_ have been chosen as the addition of polyaniline to prepare ammonia sensors, which can used at room temperature (Guo et al., 2013; Chen et al., 2015; Dai et al., 2016; Zhang et al., 2018). Most of the materials reported so far have been prepared by electrospinning, interfacial synthesis, or mechanical mixing (Talwar et al., 2014; Li et al., 2015; Nie et al., 2016). Pawar et al. synthesized PANI and TiO_2_ by chemical oxidative polymerization and sol-gel method, respectively. Then, they mixed PANI and TiO_2_ by mechanical mixing method to prepare PANI-TiO_2_ nanocomposites. The response of the sensor to 100 ppm NH_3_ is 50% (Pawar et al., 2011a). Liu et al. prepared the PANI-TiO_2_-Au ternary nanocomposite thin film by *in-situ* self-assembly method. The response of the sensor is 2.23 toward 50 ppm NH_3_, and the detection limit is 1 ppm (Liu et al., 2017). Their synthetic methods basically need more than one-step reactions. And the recovery time, sensitivity and other properties of the sensors need to be further improved.

In this work, the PANI/TiO_2_ nanocomposite was synthesized by one step hydrothermal method. The PANI/TiO_2_ nanocomposite exhibits good selectivity, sensitivity (5.4 to 100 ppm), repeatability, long-term stability, and low detection limit (0.5 ppm) to ammonia at room temperature. Meanwhile, the sensor exhibits a good liner relationship in the range of 0.5–5 and 5-100 ppm.

## Experimental

All reagents were of analytical grade, offered by Aladdin Reagent Company, and used without further purification.

### Preparation of PANI/TiO_2_ nanocomposites and pure PANI

1.147 g of ammonium persulfate (APS) was added to 10 mL of HCl (1 mol·L^−1^) to obtain solution A. In 10 mL of anhydrous ethanol, tetrabutyl titanate (TBT), and 0.47 mL of aniline were added to obtain solution B. Solution C is the mixture of 10 mL absolute ethanol and 0.47 mL aniline. The mixture solution of A and B were poured into a 50 mL Teflon-lined stainless steel autoclave and kept at 100°C for 2 h. The mixtures of solution A and C were treated as the same. The PANI/TiO_2_ nanocomposite and pure PANI was obtained after washing several times by deionized water and ethanol. With other conditions remaining constant, the amount of TBT added in solution B is 0.05, 0.1, 0.15, or 0.2 mol·L^−1^, respectively, to make products PT1, PT2, PT3, PT4. The synthetic routes of the samples are shown in Scheme S1.

### Characterization of PANI/TiO_2_ nanocomposites

The phase structure of the products were analyzed by X-ray powder diffractometer (XRD, Rigaku, D/MAX-3B) with Cu Kα1 radiation (λ = 1.54059 Å). Raman spectra of products were tested by a LabRAM HR800 laser confocal microscopic Raman spectrometer. The morphology and structure of products were investigated by scanning electron microscope (SEM, FEI/Philips, XL-30) and transmission electron microscope (TEM, Jeol, Jem-2100). Thermogravimetric analysis (TGA) was executed by a Perkin-Elmer instruments corporation thermogravimetric analyzer. The specific surface area and pore size distribution were detected by nitrogen adsorption-desorption measurement at 77 K using the TriStar II 3020 system.

### Fabrication and gas sensing measurement of the PANI/TiO_2_ nanocomposites sensors

The products were made into a paste with ethanol and coated on the surface of Al_2_O_3_ tube whose length was 4 mm and internal diameter was 0.8 mm. The Al_2_O_3_ tube brought with two gold electrodes which spaced 1 mm and the distance between two gold electrodes was 1 mm. Two platinum wires were connected with each gold electrode. After a Ni/Cr wire passed through the Al_2_O_3_ tube, it was welded to the base together with the platinum wires.

The responses of sensors were recorded by JF02E type gas sensor measurement system (Kunming Guiyan Jinfeng Technology Co. Ltd., China). The static testing method was used to measure the property of sensors. After a certain amount of NH_3_ was injected into a vacuum 10 L glass bottle, the pressure was returned to the atmospheric pressure. The gas sensor was placed in the glass bottle for testing. The sensor's response to NH_3_ was defined as S = Rg/Ra. Rg and Ra were the resistance values of the sensor in NH_3_ and air, respectively. The response time is 100s, the recovery time is defined as the time that the resistance changed 90%. The sensor response to humidity measured by the saturated solution of LiCl (11.3 RH%), CH_3_COOK (23.4 RH%), MgCl_2_ (32.8 RH%), K_2_CO_3_(43 RH%), Mg(NO_3_)_2_ (54.3 RH%), CuCl_2_ (67 RH%), NaCl (75.3 RH%), KCl (85 RH%), and KNO_3_ (93.5 RH%; Yang et al., 2017).

## Results and discussion

**Figure 1:**
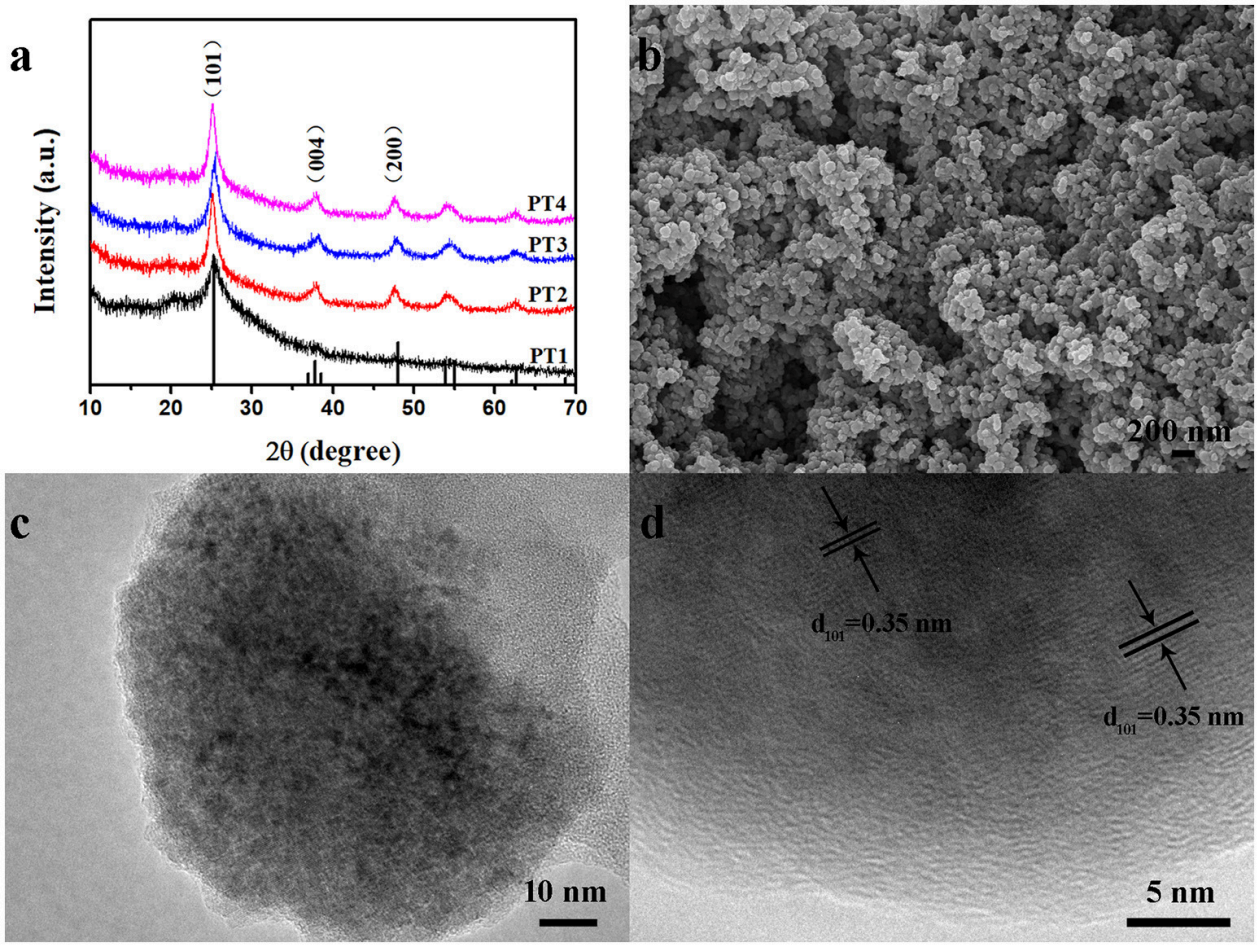
XRD patterns of the PT1, PT2, PT3 and PT4 nanocomposites **(a)**; SEM **(b)**, TEM **(c)**, and HRTEM **(d)** images of the PT3 nanocomposites.

The XRD peaks of the nanocomposites samples PT1, PT2, PT3, and PT4, shown in Figure 1a at 25°, is consistent with the XRD diffraction peak of pure PANI. As is shown in Figure S1, the diffraction peak of the pure PANI appears at about 25°. Other peaks at 25.3°, 37.8°, and 48.0° are corresponding to (101), (004), and (200) crystal planes of anatase crystal structure TiO_2_ (JCPDS Card NO.21-1272). This reminds that TiO_2_ is present in the nanocomposites. Further, the Raman spectrum of the nanocomposites was analyzed, as shown in Figure S2. The peaks at 164, 406, 635 cm^−1^ are the characteristic peaks of TiO_2_, and 1,595 cm^−1^ is attributed to the C-C stretching vibration of benzenoid ring in PANI, which further proves the presence of TiO_2_ in the nanocomposites (Chen and Mao, 2007). Through the thermogravimetric analysis (Figure S3) to determine the TiO_2_ content of PT1, PT2, PT3 and PT4 is 3.04, 19.48, 21.09, and 38.75%, respectively. The response of the PT1, PT2, PT3, PT4 nanocomposites and pure PANI to 20–100 ppm NH_3_ at room temperature is shown in Figure S4. As can be seen from the figure, the PT3 nanocomposites sensor has better response to NH_3_ than the other four sensors. Figure S5 exhibits the pore structure and specific surface area of the PT1, PT2, PT3, and PT4 nanocomposites, which were detected by N_2_ adsorption-desorption measurement and Barrett-Joyner-Halenda (BJH) pore size distribution analysis. The BET specific surface area of the PT1, PT2, PT3, and PT4 nanocomposites are 26.76, 46.13, 47.73, and 55.80 m^2^·g^−1^, respectively. The result shows that the specific surface area increase as the TiO_2_ content rises. The gas-sensing performance of the sensor is generally related to the micro-structure of sensing material, specific surface area and so on. Compared with other four sensors, the morphology of the PT3 nanocomposites is uniform (Figure S6), many TiO_2_ nanoparticles are uniformly dispersed on the surface of the PANI nanospheres, which makes the nanocomposites have many p-n heterostructure and a large specific surface area. These will increase the contact area of PT3 nanocomposites, make the NH_3_ molecules diffuse easily and provide more active sites for the efficient adsorption of NH_3_, which is more conducive to the reaction of the nanocomposite with NH_3_. Therefore, a more detailed characterization of the PT3 nanocomposites was performed.

The morphology and structure of the PT3 nanocomposites were tested by SEM and TEM. In Figure 1b, it is found that the PT3 nanocomposites were composed by the stacking of nanoparticles whose diameter is about 80 nm. Figure 1c indicates the same result that the PT3 nanocomposites is composed of nanoparticles, stacked on top of each other. In addition, the HRTEM image of the PT3 nanocomposites (Figure 1d) shows characteristic lattice fringes of TiO_2_ with a pitch of 0.35 nm, corresponding to the (101) crystal plane of TiO_2_. This is consistent with the XRD result that there is TiO_2_.

**Figure 2:**
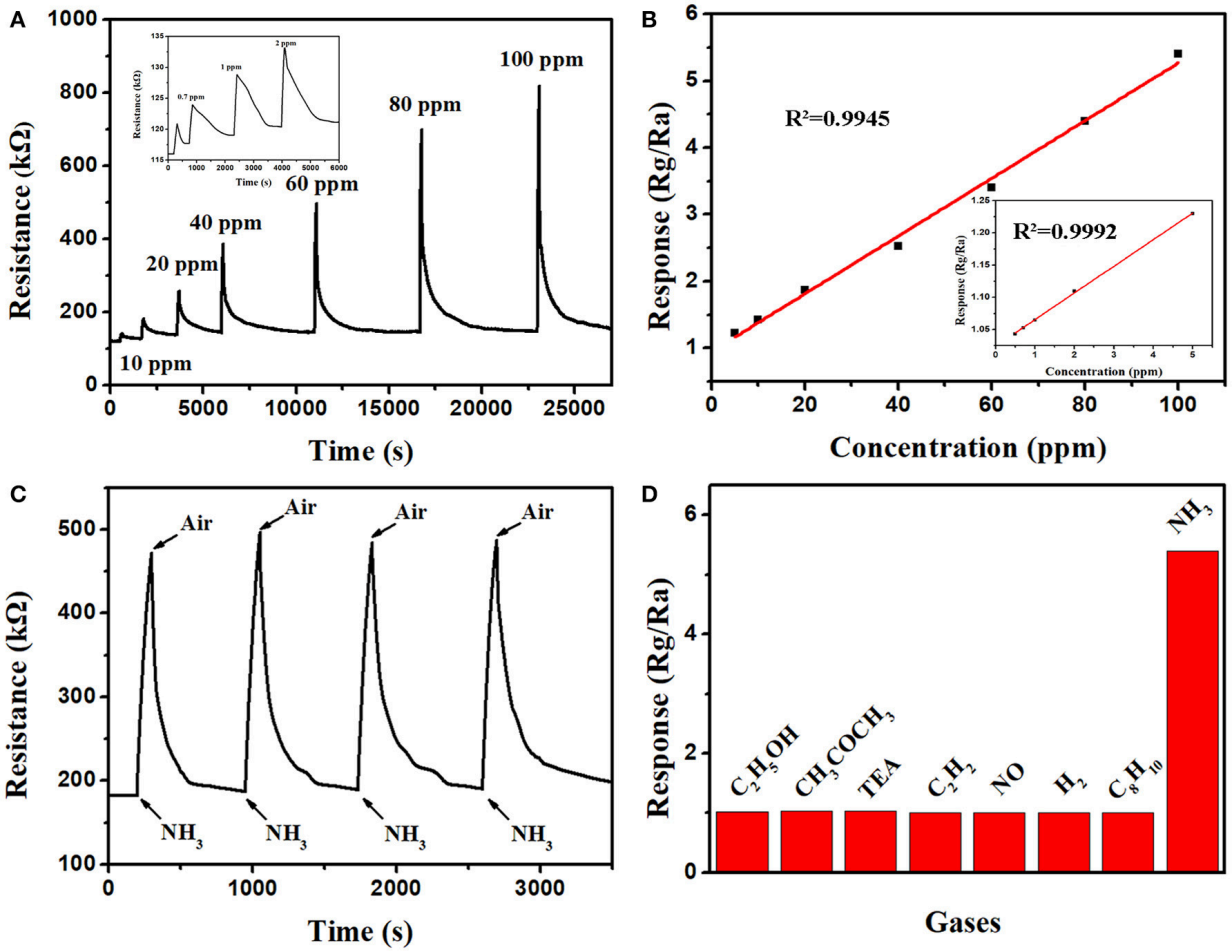
The response-recovery curves **(A)** and the linear relationship **(B)** of the PT3 nancomposites sensor to different concentrations of NH_3_; the reproducibility of the PT3 nancomposites sensor to 50 ppm NH_3_ **(C)**; the selectivity of the PT3 nanocomposites sensor to 100 ppm of eight gases **(D)**.

The gas sensing properties of the PT3 nanocomposites sensor was further studied. The response-recovery curves for 0.5–100 ppm NH_3_ at room temperature are shown in Figure 2A. To 100 ppm NH_3_, the sensitivity of PT3 nanocomposite sensor is 5.4, the response time is 100 s, and the recovery time is 232 s. Moreover, the sensitivity of the PT3 nanocomposite sensor presents a clear linear relationship with the NH_3_ concentration between 0.5 and 5 ppm (*R*^2^ = 0.9992) and 5 and 100 ppm (*R*^2^ = 0.9945) (Figure 2B). The PT3 nanocomposite sensor performed four consecutive tests on 50 ppm of NH_3_ at room temperature (Figure 2C). The corresponding sensitivities are 2.59, 2.6, 2.56, and 2.56, respectively. And the relative deviation is 1.75%. This shows that the PT3 nanocomposite sensor has a satisfactory reproducibility. The interference of other seven gases to the sensor was further researched (Figure 2D). The responds at room temperature to 100 ppm ethanol (C_2_H_5_OH), acetone (CH_3_COCH_3_), triethylamine (TEA), ethyne (C_2_H_2_), NO, H_2_, styrene (C_8_H_8_), and NH_3_ are 1.020, 1.034, 1.027, 1.006, 1.008, 1.007, 1.010, and 5.423, respectively. The result proves that the PT3 nanocomposite sensor is more sensitive to NH_3_ at room temperature. The influence of humidity was studied in the humidity range of 11.3–93.5% at room temperature (Figure S7a). The maximum response of the sensor is 1.4. It means that the effect of humidity to the sensor is much small. In order to deeply investigate the long-term stability, the sensor responds to 50 ppm NH_3_ every 5 days at room temperature. During the 2 months monitoring time (Figure S7b), the response of the sensor was reduced by < 2% after 60 days. Our investigation leads us to conclude that the sensor has a good long-term stability.

Compared the gas sensing property between the PANI-TiO_2_ nanocomposites sensor with the reported sensor in Table 1. The results show that the PT3 nanocomposites sensor exhibits excellent sensitivity and good response to NH_3_ gas at room temperature. The detection limit of PANI-TiO_2_ nanocomposites sensor is lower and sensitivity is better than the other reported sensors. The improvement of the NH_3_ sensing property of PT3 sensor is might attributed to the following reasons. First, One-step synthesis is more conducive to the dispersion of titanium dioxide nanoparticles on the surface of PANI. Secondly, the p-n heterostructure formed between TiO_2_ and PANI can provide a synergistic effect, which can effectively improve the ability to adsorb NH_3_. Therefore, the PT3 nanocomposites has potential application value in detecting NH_3_ at room temperature.

**Table 1:** Summary of recent publications of PANI-TiO_2_ nanocomposities based NH_3_ sensors.

**Sensing materials**	**Detection limit**	**Response/NH_3_ concentration**	**Response time (s)**	**Recovery time (s)**	**References**
PANI/TiO_2_	45 ppb	38.3/10.5 ppm	600	–	Li et al., 2015
PANI-TiO_2_-Au	1 ppm	2.23/50 ppm	122	–	Liu et al., 2017
PANI/TiO_2_	23 ppm	< 6/94 ppm	2 s	25 s	Tai et al., 2008
PA6/TiO_2_/PANI	–	2.6/250 ppm	150 s	450 s	Pang et al., 2014
cellulose/TiO_2_/PANI	10 ppm	3.57/100 ppm	< 150 s	800 s	Pang et al., 2016
PANI/TiO_2_	25 ppb	>0.8/200 ppb	80 s	–	Li et al., 2011
PANI/TiO_2_	–	48%/100 ppm	40 s	70 s	Pawar et al., 2012
CSA Doped PANi- TiO_2_	20 ppm	0.75/100 ppm	49 s	413 s	Pawar et al., 2011b
PANi-TiO_2_	20 ppm	50%/100 ppm	40 s	70 s	Pawar et al., 2011a
TiO_2_-SiO_2_/PANI	10 ppm	23/100 ppm	< 500 s	–	Pang et al., 2018
TiO_2_-PANI/PA6	–	18.3/250 ppm	250 s	–	Wang et al., 2012
PANi-TiO_2_	–	3.9/60 ppm	35 s	140 s	Bairi et al., 2015
PANI/TiO_2_	0.5 ppm	5.4/100 ppm	100 s	232 s	This work

The possible sensing mechanism of the PT3 nanocomposites sensor for detecting NH_3_ is as shown in Figure S8. TiO_2_ is an n-type semiconductor with a 3.2 eV forbidden band width. And PANI is a p-type semiconductor with a 2.8 eV forbidden band width. At the contact interface, the TiO_2_ and PANI interact to form a p-n heterostructure. The p-n heterostructure will make a positively charged depletion layer. So, the activation energy and enthalpy of physisorption for NH_3_ will reduce as a result to cause the enhancement of gas sensitivity (Costello et al., 1996; Tai et al., 2010). In addition, the LUMO level of PANI and the conduction band of TiO_2_ are well helps charge transfer. It can also effectively improve the gas sensing performance (Li et al., 2006; Tai et al., 2008). When the sensor is exposed to NH_3_, H^+^ on -NH- site of PANI combines with NH_3_, cause the electron hole concentration in the PANI to be low. The resistance of the sensor will increase. When the sensor is exposed to air after NH_3_, PANI gets H^+^ from ${\text{NH}}_{4}^{+}$, the electron hole concentration of PANI recovers. The resistance decreases to the initial value (Figure S8b).

## Conclusion

In summary, the PANI/TiO_2_ nanocomposites, which is consisted of uniform nanoparticles, are synthesized by one-step hydrothermal synthesis. At room temperature, the sensor based on PANI/TiO_2_ nanocomposites has a good linear relationship (0.9945 to 5–100 ppm), high response to NH_3_ (5.4 to 100 ppm), and the detection limit is 0.5 ppm. The response and recovery time to 100 ppm NH_3_ is 100 and 232 s, respectively. With small humidity effects, the sensor exhibits excellent selectivity, good reproducibility and long-term stability to NH_3_. Moreover, the excellent gas sensing property of PANI/TiO_2_ nanocomposites can be rewarded to p-n heterostructure. The more charge transfer on the surfaces where PANI is in contact with TiO_2_, the beter gas-sensing performance of PANI/TiO_2_. These results illustrate that the PANI/TiO_2_ nanocomposites sensor has great potential application for detecting NH_3_ at room temperature.

## Author contributions

CZ performed the experiments, analyzed the data with the help from YX and XC. XD wrote the manuscript with input from all authors. YX conceived the study. All authors read and approved the manuscript.

### Conflict of interest statement

The authors declare that the research was conducted in the absence of any commercial or financial relationships that could be construed as a potential conflict of interest.
