# Supplementary material for: Enhanced Sub-ppm NH3 Gas Sensing Performance of PANI/TiO2 Nanocomposites at Room Temperature
Source: Front Chem. 2018 Oct 18;6:493. doi: 10.3389/fchem.2018.00493 (PMC6201155; doi:10.3389/fchem.2018.00493)
Supplement: Supplementary file 1 [file Presentation_1.PDF]

Supplementary Material

Enhanced sub-ppm NH_3 Gas Sensing Performance of $PANI/TiO_2$ nanocomposites at room temperature

Chong-hui Zhu, Xiao-li Cheng, Xin Dong* , Ying-ming Xu*

Key Laboratory of Functional Inorganic Material Chemistry, Ministry of Education, School of Chemistry and Materials Science, Heilongjiang University, Harbin 150080, People's Republic of China.

*Correspondence: (Y. X.); (X. D.)

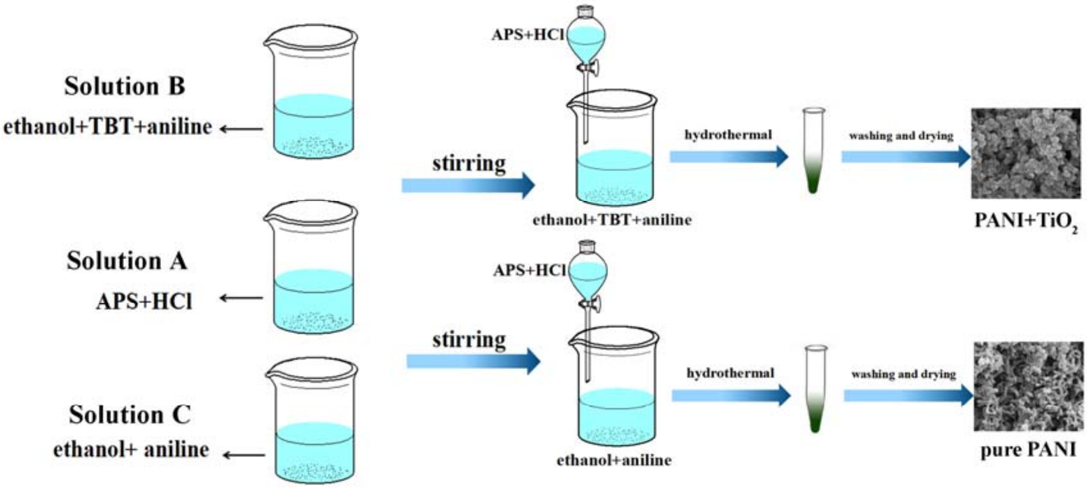

Scheme S1 The preparation schematic of $PANI-TiO_2$ and pure PANI

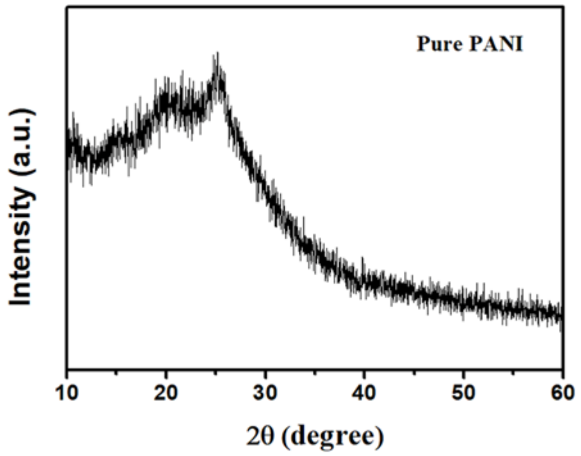

Figure S1 XRD patterns of pure PANI

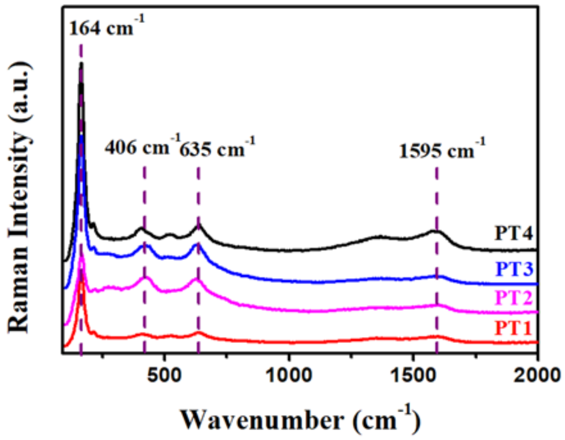

Figure S2 Raman patterns of the nanocomposites

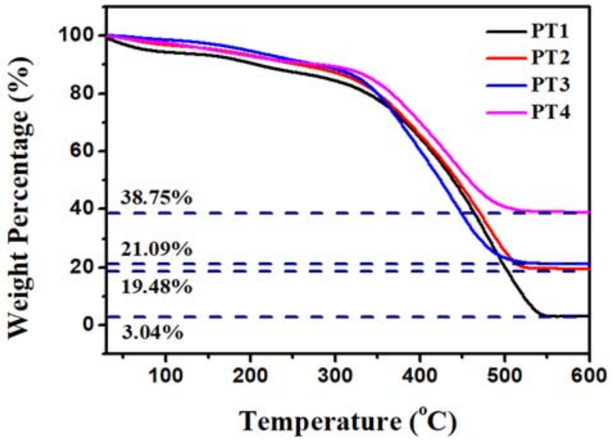

Figure S3 TG curves of the nanocomposites PT1, PT2, PT3 and PF4

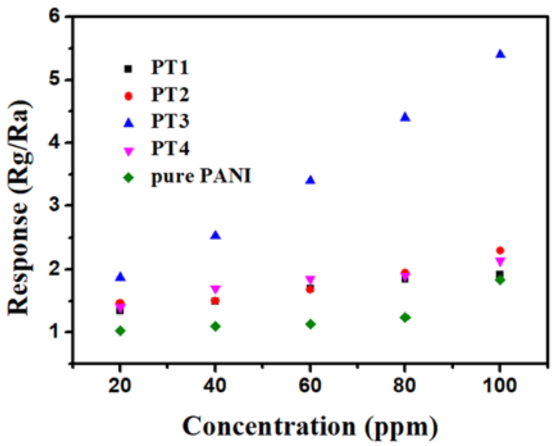

Figure S4 The sensitivities of the nanocomposites and pure PANI for 20-100 ppm NH_3 at room temperature

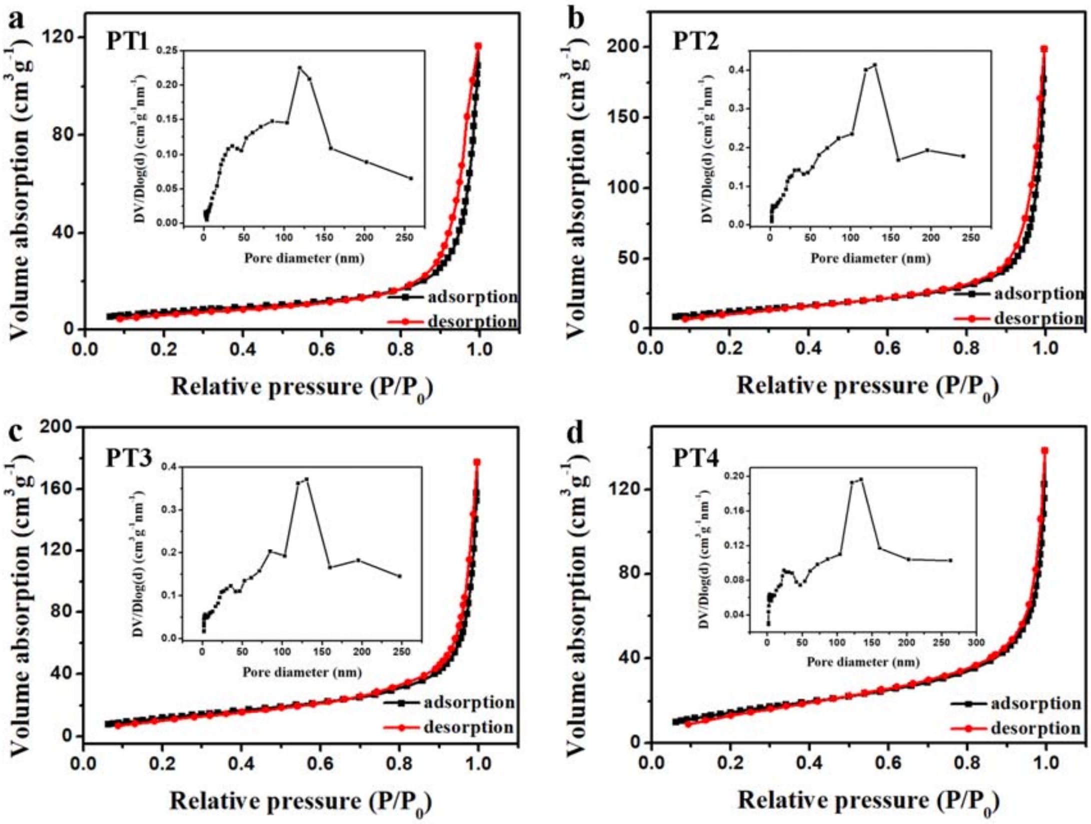

Figure S5 Nitrogen adsorption-desorption isotherms and pore diameter distribution curves of the PT1 (a), PT2 (b), PT3 (c)and the PT4 (d) nanocomposites

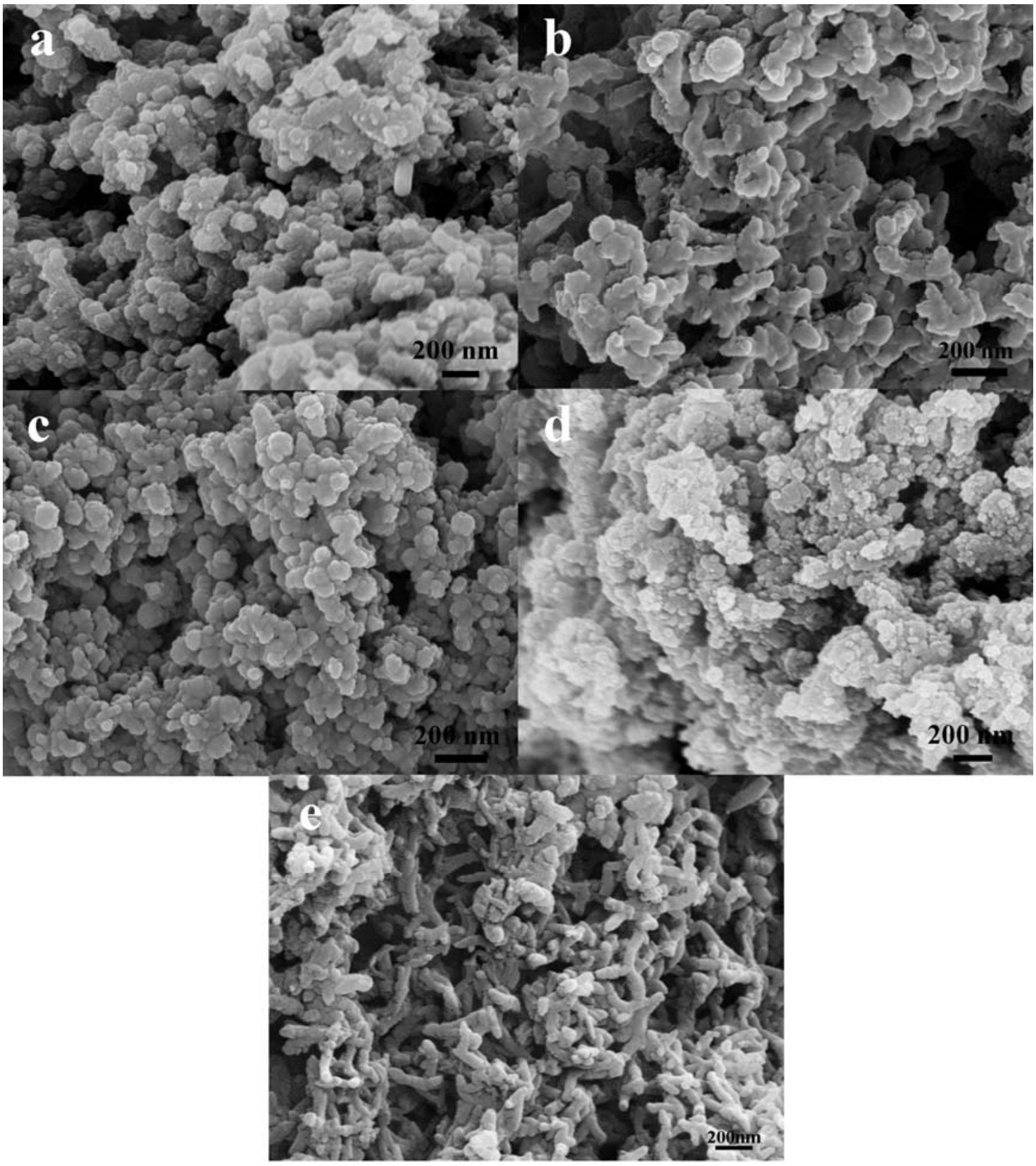

Figure S6 SEM images of nanocomposites PT1 (a); PT2 (b); PT3 (c); PT4 (d) and pure PANI (e)

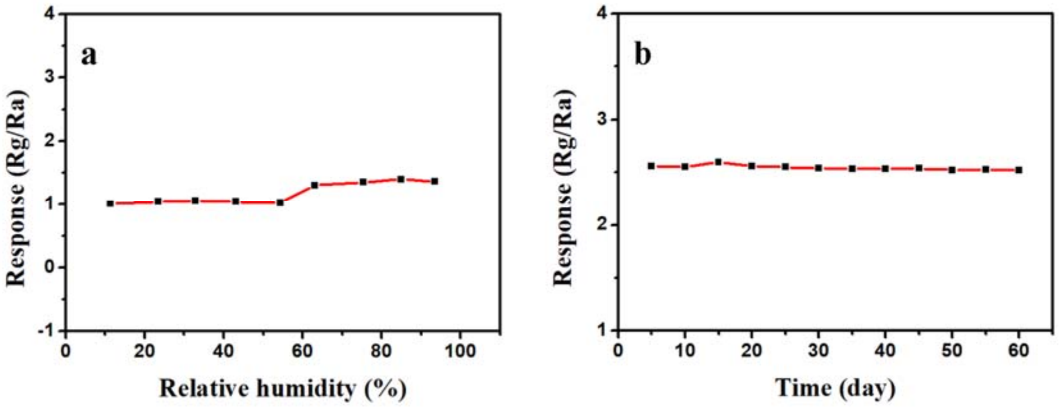

Figure S7 The response of the PT3 nanocomposities to different relative humidity at room temperature (a); the long - term stabilityof the PT3 nanocomposites sensor to 50

ppm NH_3 (b)

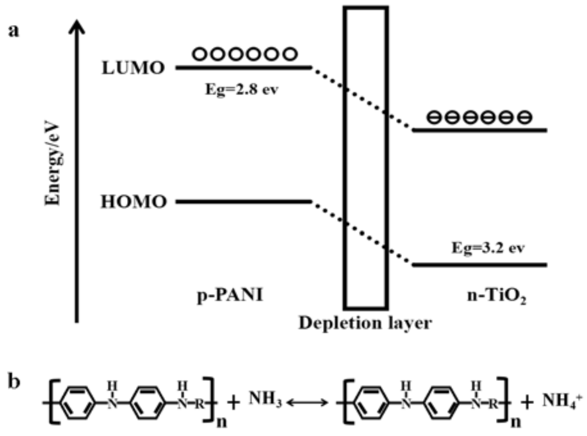

Figure S8 The formation of p-n heterojunction and energy band diagram in the PANI/TiO_2 nanocomposites (a); the possible reaction mechanism of PANI/TiO_2 nanocomposites sensor with NH_3
